# Patient-Centred Care for Older Patients Considering Surgery: An Evaluation of the Perioperative Care of Older Patients Service at an Australian Tertiary Hospital

**DOI:** 10.3390/geriatrics11030055

**Published:** 2026-04-28

**Authors:** Rachel Aitken, Katherine Gregorevic, Michelle Preeo, Ross Bicknell, Alyssa Griffiths, Jared Tower, Ned Douglas, Chuan-Whei Lee, Janette Wright, Jai Darvall, Wen Kwang Lim

**Affiliations:** 1 Department of Aged Care, Royal Melbourne Hospital, Parkville, VIC 3052, Australia; rachel.aitken2@mh.org.au (R.A.); katherine.gregorevic@mh.org.au (K.G.); michelle.preeo@mh.org.au (M.P.); ross.bicknell@mh.org.au (R.B.); 19322048@students.latrobe.edu.au (A.G.); 2 Department of Allied Health, Royal Melbourne Hospital, Parkville, VIC 3052, Australia; jared.tower@mh.org.au; 3 Department of Anaesthesia and Pain Management, Royal Melbourne Hospital, Parkville, VIC 3052, Australia; ned.douglas@mh.org.au (N.D.); chuan-whei.lee@mh.org.au (C.-W.L.); janette.wright@mh.org.au (J.W.); jai.darvall@mh.org.au (J.D.); 4 Department of Critical Care, The University of Melbourne, Parkville, VIC 3052, Australia; 5 Department of Medicine, The University of Melbourne, Parkville, VIC 3052, Australia

**Keywords:** perioperative medicine, shared decision making, frailty, comprehensive geriatric assessment

## Abstract

**Background/Objectives**: As mounting numbers of older people consider surgery, the importance of aligning treatments with patient values and goals is paramount. This has led to the growth of POPS (Perioperative care of Older Patients) services internationally and across Australia. An observational pilot evaluation of the Melbourne Health POPS service was conducted throughout 2022, with the aims of describing the population, measuring patient-reported outcomes and comparing postoperative outcomes to a matched historical cohort. **Methods**: Data were sourced from clinical review, electronic medical records and health intelligence. Patients who pursued surgery were matched 2:1 with a 2020 control cohort on up to 10 characteristics ranked on clinical judgement. Patient-reported outcomes were collected at 3 months post-surgery or at the clinic in consenting participants. **Results**: There were 128 participants, of whom 64 (50%) pursued non-surgical management. Participants were older (median 79 [13] years), frail (median CFS 5 [2]), and multimorbid (median CCI 5 [2.25]). Despite increased perioperative risk amongst the POPS surgical group (ASA-4 23.4% vs. 5.5%, *p* < 0.001), increased incidence of postoperative delirium (15% vs. 5.8%, *p* = 0.042) and ICU admission (21.7% vs. 7.5%, *p* = 0.006) compared to the control group, median length of stay was similar (4.3 [6.7] vs. 4.3 [5.1] days, *p* = 0.537). Patient-reported outcomes were similar between surgical and non-surgical POPS groups (90.7% vs. 88.1% would make the same surgical decision, *p* = 0.697). **Conclusions**: Patients attending POPS were multimorbid with geriatric syndromes and elevated perioperative risk. A high proportion pursued non-operative care. Patient-reported feedback was high with low decisional regret.

## 1. Introduction

Older people are increasingly undergoing elective surgery year on year for the purposes of longevity and symptom relief. This observation is expected to increase into the future due to an ageing population living longer with morbidity amenable to surgery, advances in perioperative care, and evolving community expectations regarding healthcare provision in later life [1,2,3]. Older people acquire age-related physiological changes [4] and are more likely to exhibit geriatric syndromes, including frailty, multimorbidity, polypharmacy, and cognitive and functional decline [5]. It is, therefore, not surprising that older patients are more likely to die and suffer medical complications and functional decline after surgery, resulting in prolonged recovery and an increased likelihood of requiring supported accommodation on discharge [6,7].

**Figure 1:**
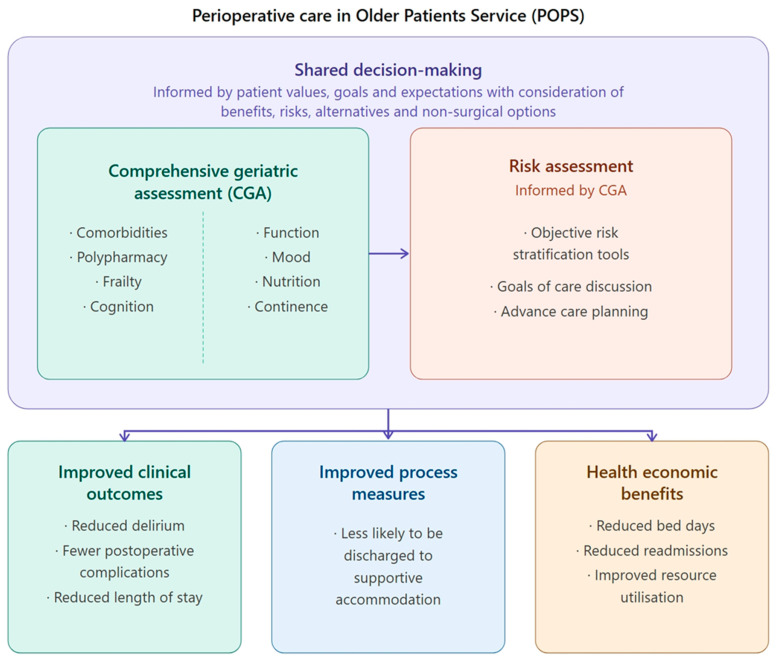
Perioperative care of Older Patients Service. Created with the assistance of Claude Sonnet 4.6 (Anthropic, 2025), USA (San Francisco, CA, USA) [16].

The specialised needs of older surgical patients have been increasingly recognised over the past decade, leading to the introduction of new models of care, establishment of special interest perioperative groups, and exponential publication of relevant research and guidelines [8,9]. Many Australian service models have taken learnings from the well-established Perioperative care of Older Patients undergoing Surgery (POPS) service based at Guy’s and St Thomas’ Hospital in London [1]. POPS utilises Geriatrician-led Comprehensive Geriatric Assessment (CGA) and management in the perioperative setting, which can be defined as a patient-centred and individualised multidimensional assessment including the evaluation and optimisation of medical comorbidities, medications, social history, function, cognition, mood and nutrition [10,11]. Shared decision-making is a key overarching principle applied within this approach, whereby estimated benefits and risks of surgery and its alternatives can be considered based on patient values and goals (Figure 1) [1,8,9,10,11].

A randomized control trial of POPS in 176 older elective vascular patients found preoperative CGA led to reduced hospital length of stay (geometric mean 3.3 vs. 5.5 days, *p* < 0.001), postoperative delirium (11% vs. 24%, *p* = 0.018), constipation (28% vs. 48%, *p* = 0.026), and cardiac complications (8% vs. 27%, *p* = 0.001), with a trend of reduced likelihood of discharge to supported accommodation (5% vs. 13%, *p* = 0.051) [12]. Subsequent economic evaluation of this trial also demonstrated cost-effectiveness [13]. In addition to RCT data, many observational studies and systematic reviews of perioperative CGA for older patients have demonstrated positive clinician-reported outcomes (Figure 1) [14,15].

Given these favourable published outcomes and an identified growing local need, the Melbourne Health POPS clinic was implemented in early 2021. The initial team consisted of a Geriatrician, Anaesthetist, and Physiotherapist conducting a one-hour or more comprehensive review. Patients from all surgical subspecialties, considering all procedures, were welcomed. Older patients aged 65 years and older were targeted; however, younger patients with geriatric syndromes or with perceived benefit were accepted. The POPS clinic was awarded a hospital Health Services Improvement Grant, which funded a research assistant to aid data collection for a pilot evaluation of the service in 2022.

The study aims were to, firstly, describe the Melbourne Health POPS cohort, secondly, to compare clinician-reported outcomes in those who pursued surgery to a historical matched pre-POPS group, and thirdly, to evaluate 3-month follow-up patient-reported outcomes.

## 2. Materials and Methods

A prospective observational cohort study of a POPS pilot group was conducted at the Royal Melbourne Hospital, Victoria, Australia.

### 2.1. Setting and Participants

The POPS clinic is a geriatrician-led multidisciplinary clinic providing preoperative comprehensive geriatric assessment for predominantly older, multimorbid patients who commonly have geriatric syndromes such as frailty and cognitive and functional impairment. All patients who attended the POPS clinic for consideration of elective surgery were included in the study as part of usual care during the recruitment period, March–December 2022. A waiver of consent was obtained for all POPS attendees, and verbal consent for a 3-month follow-up phone call was discussed and documented during the clinic.

### 2.2. Data Collection

Preoperative demographic, clinical, perioperative, and patient-reported data were collected at the POPS clinic by the geriatrician, anaesthetist, and physiotherapist team, supported by a research assistant. Where telehealth was necessary in a minority of cases, cognitive and bedside functional tools were incomplete. Perioperative process measures and postoperative outcomes for those who pursued surgery were collected from electronic medical records. Length of stay was calculated with assistance from the hospital Health Intelligence Unit. Consenting patients also received a follow-up phone call 3 months after surgery or the final POPS clinic in those who chose non-surgical management, for the purposes of collecting patient-reported quality of life and experience data. All data were stored in a secure REDCap database.

### 2.3. Matched Control Group

**Figure 2:**
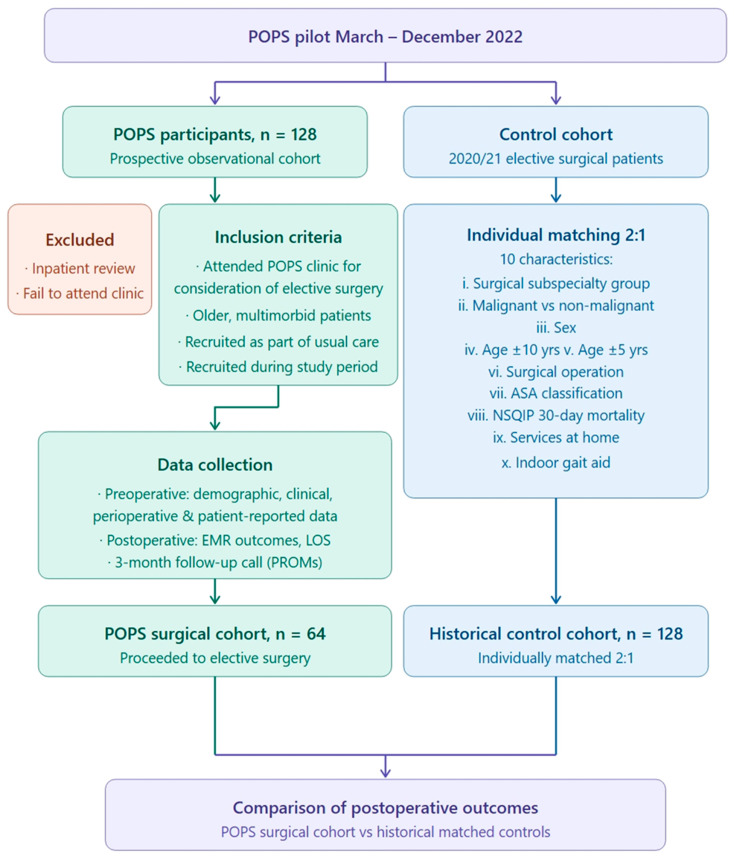
Schematic representation of study design. Created with the assistance of Claude Sonet 4.6 (Anthropic, 2025) [16].

A historical matched control group was sought for comparison of perioperative process measures and clinician-reported outcomes. Controls were individually matched 2:1 via manual selection from a list of 2020 elective surgical patients provided by the Health Intelligence Unit. Matching was performed using 10 characteristics ranked in order of perceived clinical importance: (i) surgical subspecialty group; (ii) malignant vs. non-malignant surgical issue; (iii) sex; (iv) age within 10 years; (v) age within 5 years; (vi) surgical operation (vii) American Society of Anaesthesiologists Classification (ASA); (viii) National Surgical Quality Improvement Program (NSQIP) 30 day mortality; (ix) services at home; (x) indoor gait aid (Figure 2).

### 2.4. Outcome Measures

The primary outcome of this study was the proportion of POPS patients pursuing a non-surgical management pathway. Secondary outcomes comprised surgical vs. non-surgical patient-reported outcomes at 3 months and clinician-measured outcomes in surgical POPS patients compared to the historical matched cohort, including mortality, complications, length of stay, and discharge destination.

### 2.5. Statistical Analysis and Ethics

Descriptive statistical analysis was performed using JASP (Version 0.95.3) software [17]. The Chi-square test was used to compare categorical variables, whereas the Mann–Whitney U test was used to compare continuous non-parametric data using median and interquartile range. Statistical significance was defined as *p* < 0.05 for all analyses. This protocol was assessed by the Royal Melbourne Hospital Human Research Ethics Committee as appropriate for a quality assurance project and assigned reference QA2021131, approved 9 February 2022. Infographic Figure 1 and Figure 2 were created with assistance from Claude Sonnet 4.6 (Anthropic, 2025), USA (San Francisco, CA, USA) a generative AI tool. Both figures were reviewed and edited by the authors for accuracy.

## 3. Results

### 3.1. Demographics and Clinical Characteristics

There were 128 participants who attended the POPS preoperative clinic throughout the data collection period. The cohort was older (median age 79 [13]) and more likely to be male (59.4%) and have English as a second language (56.3%), such that 32.8% used an interpreter. A high proportion used a gait aid (outdoor gait aid 62.5%), and 10.9% resided in supported accommodation. The POPS group were highly comorbid (CCI median 5 [2.25]) and frail (median CFS 5 [2]). A total of 11.7% had diagnosed dementia and 26.6% a history of delirium, with poor median functional reserve (DASI 9.95 (12.6)) and timed up and go 17.3 [11.2] seconds. This correlates with the high observed proportion of ASA-3 (69.5%) and ASA-4 (25.8%) classification (Table 1).

### 3.2. Perioperative Process Measures

The POPS cohort included a wide spectrum of nine or more surgical specialties, of which gastrointestinal (31.3%) and orthopaedic (25.3%) were the most common. A total of 23.4% of patients were considering surgery for the purpose of cancer treatment. A total of 73.4% received a single POPS preoperative review, and 23.4% underwent two reviews. Overall, 50% ultimately pursued a non-operative management strategy. A total of 4 (6.3%) made this decision pre-POPS, 32 (50.8%) during the period of POPS assessment, and 27 (43.9%) post-POPS.

### 3.3. Control Group Matching

The 2020 control cohort was well matched at a 2:1 ratio on the majority of 10 nominated variables, including age, sex, operation type, and home supports (see Table S1). Despite this, the control group was markedly less comorbid (CCI 4 [1] vs. 5 [2], *p* = 0.002), had a lower number of medications (7 [4.3] vs. 9 [7], *p* < 0.001), was less likely to have dementia (0.8% vs. 6.3%, *p* = 0.025), and was less likely to be ASA-4 (5.5% vs. 23.4%, *p* < 0.001), conferring significantly lower control group perioperative risk.

### 3.4. Clinical Outcomes in POPS Surgical Cohort

A total of 60 POPS patients from the original cohort proceeded to surgery at the Royal Melbourne Hospital, whereas 4 had surgery elsewhere, such that clinician-measured outcomes were unavailable. Within these 60, a higher rate of postoperative complications measured by Clavien–Dindo scale (CD ≥ III 15% vs. 5.8%, *p* = 0.042) and postoperative delirium (15% vs. 5.8%, *p* = 0.042) was observed compared to the matched control group. POPS patients were also more likely to require postoperative ICU admission (21.7% vs. 7.5%, *p* = 0.006) and readmission to the hospital within 30 days (18.3% vs. 3.3%, *p* < 0.001). Despite these findings, acute length of stay was statistically similar between both groups (median 4.26 [6.71] vs. 4.25 [5.07] days, *p* = 0.537), and results must be interpreted with acknowledgement of residual study and control group perioperative risk differences (Table 2).

### 3.5. Patient-Reported Outcomes

Health-related quality of life scores were moderate at baseline with a median EQ-5D-5L index of 0.65 [0.47] and a median self-rated health visual analogue scale (VAS) of 50 [35] (Table 3). There was minimal change at the time of follow-up across both surgical and non-surgical groups, although a trend towards better results in the surgical group was observed. The surgical cohort also had a higher Katz measure of functional independence on follow-up (median 6 [1.7] vs. 5 [3], *p* = 0.017); however, similar proportions were residing at home. When patients were asked to reflect on their experience attending POPS and their subsequent perioperative journey, including decisional regret, the value of POPS and overall perioperative experience, ratings were generally high and similar between both follow-up groups (see corresponding results in Table 3). Informal subjective feedback reported to the research assistant ranged between trouble remembering the POPS clinic, finding explanation of risk assessment unpleasant and unhelpful, and high satisfaction and engagement in shared decision-making, with many stating they appreciated the time spent listening to their values and needs.

## 4. Discussion

This observational pilot study sought to evaluate the recently implemented Melbourne Health POPS service, including a comprehensive description of the cohort with cognitive screening, physiotherapy-led bedside functional tests, perioperative process measures, and patient-reported outcomes. The patient cohort can be identified as a highly vulnerable group characterised by advanced age, multimorbidity, frailty, and functional impairment, reflective of the complexity of patients increasingly encountered in contemporary elective surgical practice. A high proportion of patients cited a culturally and linguistically diverse background (56.3% English second language), which poses further healthcare provision challenges. High rates of cognitive vulnerability (11.7% dementia; 26.6% prior delirium), polypharmacy (median 9 [6] medications), impaired mobility (62.5% using an outdoor gait aid), and elevated anaesthetic risk (>95% ASA 3–4) additionally highlight the potential benefit of a geriatrician-led multidisciplinary comprehensive geriatric assessment (CGA) model in this setting. The striking result of 50% of patients choosing to pursue a non-operative pathway is higher than figures previously described in local and international literature [18]. Explanations for this incongruence are beyond the scope of this study; however, it is suspected that the selection of a markedly frail and comorbid population compared to what has been described elsewhere has contributed. It also suggests that dedicated risk assessment and shared decision-making aimed at aligning treatment with individual values can significantly impact patient outcomes.

Despite careful individual matching on ten clinically relevant variables, the historical control group was less comorbid, was less frail, and had lower perioperative risk profiles, including lower proportion of ASA-4 (5.5% vs. 23.4%, *p* < 0.001) and predicted NSQIP 30-day mortality (0.5 vs. 1.3%, *p* < 0.001) compared to the POPS surgical cohort. Factors hypothesised as contributing to this finding include the change in surgical practice during the COVID-19 pandemic in 2020, impacting the control group data, and a markedly frail and comorbid group referred to the recently implemented POPS service post-COVID. When interpreted in this context, the comparable median acute length of stay between the groups (4.3 [6.7] vs. 4.3 [5.1] days) may in fact represent a relative success, as one might otherwise predict a longer admission in the more comorbid POPS cohort. Similarly, rates of inpatient mortality and discharge to supported accommodation were relatively low and not statistically different between the two groups. In contrast to findings from earlier UK-based studies of preoperative CGA [12,15], we did not observe reductions in delirium or major complications, and instead, the POPS cohort exhibited higher rates of delirium, Clavien–Dindo ≥III complications, ICU admission, and 30-day readmission. Again, the impact of selection bias towards a frail, comorbid group with high functional and cognitive burden (11.7% dementia; 26.6% prior delirium) must be considered when interpreting this result. Overall, these mixed findings emphasise the complexity of evaluating geriatric perioperative services using conventional surgical outcome measures alone.

Attempts to describe patient-reported outcomes in the highly culturally and linguistically diverse, cognitively impaired and vulnerable, and unwell POPS population were challenging, reflected by the low data completion rate. However, this aspect of study design was thought to offer highly valuable insights into patient symptom burden, quality of life, and experience and hence pursued. At three months, the majority of patients remained living at home, and health-related quality of life as measured by EQ-5D-5L was stable or modestly improved, particularly amongst those undergoing surgery. Importantly, nearly 90% of all POPS patients reported they would make the same surgical decision again, and over 80% perceived that POPS added value to their care, with high overall perioperative experience ratings (median 9/10). As might be predicted, the surgical group had a higher functional state at three months compared to the non-operative group (median Katz 6 [1.7] vs. 5 [3], *p* = 0.017). These findings serve as a reminder that patient-centred metrics, including decisional satisfaction and perceived value, can capture the benefits of preoperative CGA in addition to clinician measures alone.

This study had several limitations that should be considered when interpreting the findings. Firstly, the observational design and relatively small sample size reduce statistical power to detect differences in clinically significant infrequent outcomes such as mortality and major complications. Secondly, there is potential for selection bias given the non-standardised referral pathway to POPS targeting those with a higher perceived perioperative risk. In addition, the inherently heterogeneous POPS group and intervention with variability in number of consultations, disciplines involved, and optimisation strategies employed limit the ability to attribute observed outcomes to specific components of care. Patient-reported outcome data were incomplete and subject to response bias, compounded by language barriers, cognitive impairment, health literacy, medical illness, and lengthy times awaiting surgery in some cases. Finally, the generalizability of the findings observed in this single-centre tertiary hospital service may not extend to other healthcare settings.

Future research aimed at evaluating the POPS model of care should remain prospective, include an increased number of older patients, and ideally use contemporaneous comparison groups using matched objective risk assessment tools such as frailty. Given the high proportion of patients who pursued non-operative management in this pilot study, further investigation into this cohort is warranted, including clinical trajectory, healthcare utilisation, and longer-term quality of life measures. Ongoing emphasis on patient-reported outcomes, including functional independence, discharge destination, and decisional regret, is a vital component of this research given the fundamental goal of aligning treatment decisions with patient values and goals. Formal health economic analysis is also required to determine the cost-effectiveness and sustainability of POPS within the Australian healthcare system.

## 5. Conclusions

This evaluation of POPS at an Australian tertiary centre demonstrates the complexity and elevated perioperative risk in older people contemplating surgery for the purposes of symptom relief and longevity. When offered comprehensive geriatric assessment, risk assessment, shared decision-making, and in some cases, optimisation, 50% of this cohort ultimately pursued non-surgical care. Matching this high-risk group to a historical cohort proved challenging and may have impacted postoperative outcomes. Patient-reported feedback was high with low decisional regret, highlighting the importance of aligning treatment with underlying values and goals.

## Figures and Tables

**Table 1:** Patient characteristics.

Covariate	POPS Cohort (n = 128)	POPS Surgical Cohort (n = 64)	Control Cohort (n = 128)	*p* Value
Age, median (IQR)	79 (13)	77 (11.5)	77 (12)	0.803
Male sex, n (%)	76 (59.4%)	39 (60.9%)	75 (58.6%)	0.755
ATSI, n (%)	1 (0.8%)	0 (0%)	4 (3.1%)	0.153
English second language, n (%)	72 (56.3%)	38 (59.3%)	36 (38.1%)	<0.001
Interpreter used, n (%)	42 (32.8%)			
Support person present, n (%)	103 (80.5%)			
Supported accommodation, n (%)	14 (10.9%)	6 (9.4%)	0 (0%)	<0.001
Indoor gait aid or immobile, n (%)	64 (50%)	29 (45.3%)	55 (44.7%)	0.938
Outdoor gait aid, n (%)	80 (62.5%)			
CCI, median (IQR)	5 (2.25)	5 (2)	4 (1)	0.002
Ischaemic Heart Disease, n (%)	42 (32.8%)	17 (26.6%)	37 (28.9%)	0.733
Arrhythmia, n (%)	43 (33.6%)	18 (28.1%)	27 (21.1%)	0.278
Heart Failure, n (%)	35 (27.3%)	12 (18.8%)	10 (7.8%)	0.025
COPD, n (%)	33 (25.8%)	15 (23.4%)	19 (14.8%)	0.141
Chronic Kidney Disease, n (%)	38 (29.7%)	21 (32.8%)	21 (16.4%)	0.01
Diabetes, n (%)	45 (35.2%)	24 (37.5%)	26 (28.1%)	0.186
No. of Medications, median (IQR)	9 (6)	9 (7)	7 (4.3)	<0.001
CFS, median (IQR)	5 (2)	5 (2)		
EFS, median (IQR)	8 (5)	8 (4.3)		
BMI, median (IQR)	27.1 (7.2)	26.8 (8.6)	28.3 (8)	0.146
MUST ≥ 2, n (%)	17 (13.3%)	8 (12.5%)		
History of Dementia, n (%)	15 (11.7%)	4 (6.3%)	1 (0.8%)	0.025
History of Delirium, n (%)	34 (26.6%)	14 (21.9%)	5 (0.39%)	<0.001
MMSE/RUDAS, median (IQR)	26 (5)	27 (4.7)		
Katz, median (IQR)	5 (2)	6 (1.3)		
DASI, median (IQR)	9.95 (12.6)	12.45 (11.7)		
TUGT, median (IQR)	17.3 s (11.2)	15.3 s (13.3)		
DEMMI, median (IQR)	57 (23)	57 (33)		
Hx of Anxiety or Depression, n (%)	36 (28.1%)	16 (25%)	41 (32%)	0.315
ASA 3, n (%)	89 (69.5%)	47 (73.4%)	98 (76.6%)	0.635
ASA 4, n (%)	33 (25.8%)	15 (23.4%)	7 (5.5%)	<0.001
NSQIP 30-day mortality, median (IQR)	1.95 (4.1)	1.3 (3.6)	0.5 (1.3)	<0.001
Orthopaedic, n (%)	33 (25.8%)			
Gastrointestinal, n (%)	40 (31.3%)			
Malignant, n (%)	30 (23.4%)	18 (28.1%)	33 (25.8%)	0.729
Surgical urgency category 1, n (%)	39 (30.5%)			
Surgical POPS referral, n (%)	75 (58.6%)			
Number of POPS preop reviews, n (%)	1 = 94 (73.4%)			
	2 = 30 (23.4%)			
	3 = 4 (3.1%)			
Non-surgical malignant, n (%)	64 (50%)			
Operation duration > 4 h		10 (16.7%)	16 (13.3%)	0.594
General anaesthesia, n (%)		52 (85%)	107 (90%)	0.325

IQR, interquartile range; ATSI, Aboriginal and Torres Strait Islander; CCI, Charlson Comorbidity Index; COPD, Chronic Pulmonary Obstructive Disease; CFS, Clinical Frailty Scale; EFS, Edmonton Frailty Scale; BMI, Body Mass Index; MUST, Malnutrition Universal Screening Tool; MMSE, Mini-Mental State Examination; RUDAS, Rowland Universal Dementia Assessment Scale; DASI, Duke Activity Status Index; TUGT, Timed Up and Go test; DEMMI, de Morton Mobility Index; ASA, American Society of Anaesthesiologists; NSQIP, National Surgical Quality Improvement Program.

**Table 2:** Clinician-measured outcomes: POPS surgical group vs. matched control group.

Outcome Measure	POPS Surgical Cohort (n = 60)	Matched Control Group (n = 120)	*p* Value
Acute hospital length of stay days, median (IQR)	4.26 (6.71)	4.25 (5.07)	0.537
Inpatient mortality, n (%)	2 (3.1%)	1 (0.8%)	0.217
Discharge destination, n (%)			
- home	41 (68.3%)	96 (80%)	0.084
- inpatient rehabilitation	12 (20%)	19 (15.8%)	0.485
Postoperative complications:			
Clavien–Dindo classification ≥ III, n (%)	9 (15%)	7 (5.8%)	0.042
Delirium, n (%)	9 (15%)	7 (5.8%)	0.042
Chest Infection, n (%)	3 (5%)	3 (2.5%)	0.378
AMI, n (%)	1 (1.7%)	4 (3.3%)	0.521
Tachyarrhythmia, n (%)	4 (6.7%)	4 (3.3%)	0.521
Heart Failure, n (%)	1 (1.7%)	3 (2.5%)	0.721
AKI, n (%)	4 (6.7%)	9 (7.5%)	0.839
UTI, n (%)	3 (5%)	4 (3.3%)	0.586
VTE, n (%)	0	1 (0.8%)	0.478
Stroke, n (%)	0	1 (0.8%)	0.478
Surgical complication, n (%)	7 (11.7%)	8 (6.7%)	0.253
Postoperative ICU admission, n (%)	13 (21.7%)	9 (7.5%)	0.006
Unplanned ICU admission, n (%)	7 (11.7%)	4 (3.3%)	<0.001
Readmission within 30 days, n (%)	11 (18.3%)	4 (3.3%)	<0.001
3-month mortality, n (%)	4 (6.3%)		

AMI, acute myocardial infarction; AKI, acute kidney injury; UTI, urinary tract infection; VTE, venous thromboembolism; ICU, Intensive Care Unit.

**Table 3:** Patient-reported outcomes.

Covariate	POPS Baseline (n = 128)	POPS Surgical Follow-Up (n = 43)	POPS Non-Surgical Follow-Up (n = 43)	*p* Value
Living at home, n (%)	114 (89%)	38 (88.4%)	37 (86%)	0.747
Katz, median (IQR)	5 (2)	6 (1.7)	5 (3)	0.017
EQ-5D-5L Index, median (IQR)	0.65 (0.47)	0.76 (0.25)	0.6 (0.52)	0.061
EQ-5D-5L VAS, median (IQR)	50 (35)	60 (37.5)	50 (35)	0.37
I would make the same surgical decision, n (%)		39 (90.7%)	37 (88.1%)	0.697
I felt POPS added value to my care, n (%)		34 (78.2%)	34 (81%)	0.446
Overall perioperative experience rating/10		9 (2)	9 (1.55)	0.81
median (IQR)				

EQ-5D-5L, EuroQol 5-dimension 5-level questionnaire; VAS, visual analogue scale.

## Data Availability

The original contributions presented in this study are included in the article/Supplementary Materials. Further inquiries can be directed to the corresponding author.

## Supplementary Materials

The following supporting information can be downloaded at: https://www.mdpi.com/article/10.3390/geriatrics11030055/s1, Table S1: Control cohort matching on 10 variables (n = 120).

## Abbreviations

The following abbreviations are used in this manuscript:

POPS	Perioperative care of Older Patients Service
CFS	Clinical Frailty Scale
CCI	Charlson Comorbidity Index
ICU	Intensive Care Unit
CGA	Comprehensive Geriatric Assessment
RCT	Randomised Control Trial
ASA	Americal Society of Anaesthesiologists Classification
NSQIP	National Surgical Quality Improvement Program
DASI	Duke Activity Status Index
IQR	Interquartile Range
ATSI	Aboriginal and Torres Strait Islander
EFS	Edmonton Frailty Scale
BMI	Body Mass Index
MUST	Malnutrition Universal Screening Tool
MMSE	Mini-Mental State Examination
RUDAS	Rowland Universal Dementia Assessment Scale
TUGT	Timed Up and Go test
DEMMI	De Morton Mobility Index
CD	Clavien–Dindo classification
AMI	Acute Myocardial Infarction
AKI	Acute Kidney Injury
UTI	Urinary Tract Infection
VTE	Venous Thromboembolism
EQ-5D-5L	EruoQol 5-dimension 5-level questionnaire
VAS	Visual Analogue Scale
