# Supplementary material for: Patient-Centred Care for Older Patients Considering Surgery: An Evaluation of the Perioperative Care of Older Patients Service at an Australian Tertiary Hospital
Source: Geriatrics (Basel). 2026 Apr 28;11(3):55. doi: 10.3390/geriatrics11030055 (PMC13214604; doi:10.3390/geriatrics11030055)
Supplement: Supplementary file 1 [file geriatrics-11-00055-s001.zip › geriatrics-4211858-supplementary.pdf]

Table S1: Control cohort matching on 10 variables (n = 120)

Study match	Surgical subspecialty	Malignant vs non-malignant	Sex	Age within 10 years	Age within 5 years	Operation	ASA	NSQIP 30-day mortality	Services at home or residential care	Indoor gait aid
1										
1										
2										
2										
3										
3										
4										
4										
8										
8										
10										
10										
11										
11										
12										
12										
13										
13										
17										
17										
18										
18										
29										
29										
31										
31										
32										
32										
35										

35										
38										
38										
40										
40										
41										
41										
44										
44										
45										
45										
48										
48										
49										
49										
50										
50										
53										
53										
55										
55										
56										
56										
57										
57										
59										
59										
60										
60										
61										
61										
62										
62										
67										
67										

71										
71										
72										
72										
75										
75										
77										
77										
80										
80										
81										
81										
82										
82										
87										
87										
89										
89										
90										
90										
91										
91										
93										
93										
94										
94										
95								Unable		
95								Unable		
96										
96										
97										
97										
98										
98										
100										

100										
104										
104										
107										
107										
108										
108										
110										
110										
113										
113										
117										
117										
118										
118										
119										
119										
122										
122										
124										
124										
127										
127										

Key:

	Matched
	Similar or partially matched
	Unmatched

NSQIP: matched defined as within 1%, partially matched defined as within 2%, >2% discrepancy classified unmatched
